# Comprehensive Review and Critical Evaluation of the Half-Life of Tritium

**DOI:** 10.6028/jres.105.043

**Published:** 2000-08-01

**Authors:** L. L. Lucas, M. P. Unterweger

**Affiliations:** National Institute of Standards and Technology, Gaithersburg, MD 20899-8462, U.S.A

**Keywords:** evaluation, half-life, hydrogen-3, review, tritium

## Abstract

As part of the preparation and calibration of three new National Institute of Standards and Technology (NIST) tritiated-water radioactivity Standard Reference Materials (SRMs), we have performed a comprehensive review and critical evaluation of the half-life of tritium (hydrogen-3). Twenty three experimentally-determined values of the half-life of tritium, reported between 1936 and 2000, were found. Six of these values were updated by later values. Two values were limits. Two values were deemed to be outliers. The 13 remaining values were evaluated in several ways. The results are compared with the results of other recent evaluations and all are found to be in good agreement. Our final recommended value for the half-life of tritium is the average of the adopted values from the four most recent evaluations, (4500 ± 8) d, where 8 d corresponds to one standard uncertainty.

## 1. Introduction

The history of tritium (hydrogen-3) is an interesting one [1]. The first measurement of the half-life of tritium was reported by McMillan [2] in 1936, more than 3 years before Alvarez and Cornog [3] reported the discovery of radioactive tritium and made their own measurements of the half-life [4,6]. McMillan measured the rate of decay of the radiation from a beryllium target that had been irradiated with deuterons for about a year in the cyclotron at the University of California at Berkeley. McMillan thought that the radiation might be from beryllium-10. It was realized several years later [5,6] that the radiation was actually from tritium.

Since that time, there have been numerous measurements of the half-life of tritium. As part of the preparation and calibration of three new National Institute of Standards and Technology (NIST) tritiated-water radioactivity Standard Reference Materials (SRM 4361C, SRM 4926E, and SRM 4927F), we have performed a comprehensive review and critical evaluation of the reported half-lives. All of the experimentally-determined values of the half-life of tritium [2,4,6–26] known to the evaluators as of March 2000 are shown in Table 1. These measurements were reported between 1936 and 2000. The 23 half-life values listed are the direct result of experimental measurements carried out by the author(s) of the cited references in the table. The most recent direct experimental measurement [26] was performed as part of the calibration of the new NIST standards. In addition, one half-life value was reported [27] (not included in Table 1) that was calculated using published experimental values of the tritium beta end-point energy, published experimental values of the heat output per gram of tritium, and a theoretically derived ratio of the average beta decay energy to the beta end-point energy.

## 2. Screening of the Data

The values shown in Table 1 were first screened. The screened values are shown in Table 2. The screening was done as follows:

- We obtained a copy of each publication and carefully read it.
- We verified the values listed in Table 1 as being the reported values.
- We examined the data presented in the publication to obtain the best value of the reported half-life in days. In most cases, the time was actually measured in days and the decay constant was actually computed in terms of reciprocal days or reciprocal seconds. Where only the half-life in years was reported, it was converted to the half-life in days (by multiplying by 365.2422 d per mean solar year). The preferred unit for the tritium half-life is the day because:
  - it is a well-defined unit, equal to 86 400 s, and the second is a unit of the International System of Units (SI);
  - it is the most appropriate unit for most calculations, since decay times are almost always actually measured in days; and
  - it eliminates the conversion and confusion associated with different “years” (calendar, solar, sidereal, etc.).
- We determined the meaning of the author’s stated uncertainty (confidence limit, probable error, standard deviation, etc.). (In some cases, it was not possible to determine the meaning of the author’s stated uncertainty.) We then calculated the author’s equivalent standard uncertainty (i.e., the author’s equivalent estimated standard deviation).
- We made an independent estimate of the standard uncertainty of the reported half-life. If the author’s equivalent standard uncertainty was within a factor of 2 of our estimate, then we used the author’s equivalent standard uncertainty. If not, we used our estimate (see Sec. 3, Reevaluation of Uncertainties).
- We determined whether the reported value updated an earlier reported value, either the half-life or the uncertainty. An earlier value was considered to be updated by a later value if the data upon which the later value was based included the data upon which the earlier value was based. When this was the case, the earlier value was omitted from further evaluation. Six values were omitted because of later updates [4,10,15,17,20,21].
- We determined whether the reported value was a limit or was an outlier. Two values are limits [2,6]. Two values are clearly outliers [7,9], each having a difference of more than 50 standard deviations from the mean of the remaining distribution. Two other values [8,14] are marginal (see Sec. 4, Test for Normality of Data). Table 2 includes these two marginal values. The statistical calculations were carried out both with and without these two values, to see if there was any significant difference in the results.

## 3. Reevaluation of Uncertainties

In an evaluation such as this, which includes values reported from 1936 to 2000 in Table 1 and from 1947 to 2000 in Table 2, the most difficult problem is to evaluate the uncertainty associated with each measurement in a consistent way. Once one has a set of consistent uncertainty estimates, the various statistical treatments can be carried out and the results of the various treatments can be meaningfully compared.

Since the mid 1980s, most authors have reported their measurement uncertainties more thoroughly and more in accord with internationally-accepted guidelines [28]. Before 1980, most authors reported uncertainties whose meanings were often unstated. Even when stated, the uncertainties varied widely for seemingly similar measurements.

Therefore, as part of this evaluation, we made an independent estimate of the standard uncertainty of each reported half-life. We recognize that there is a large uncertainty associated with each of our estimates. Hence, if the author’s equivalent standard uncertainty was within a factor of 2 of our estimate, then we used the author’s equivalent standard uncertainty. If not, then we used our estimate.

## 4. Test for Normality of Data

**Fig. 1:**
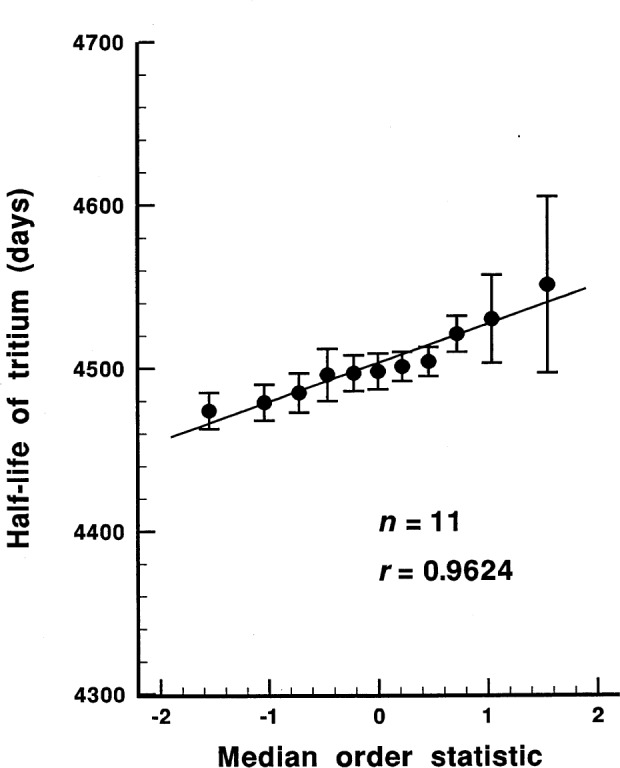
Normal probability plot for the *n* = 11 data set. The abscissa is the median order statistic from a normal *N*(0,1) distribution as given by Filliben [29]. The test statistic *r* is the normal probability plot correlation coefficient (i.e., the correlation coefficient for the linear regression line that is shown).

**Fig. 2:**
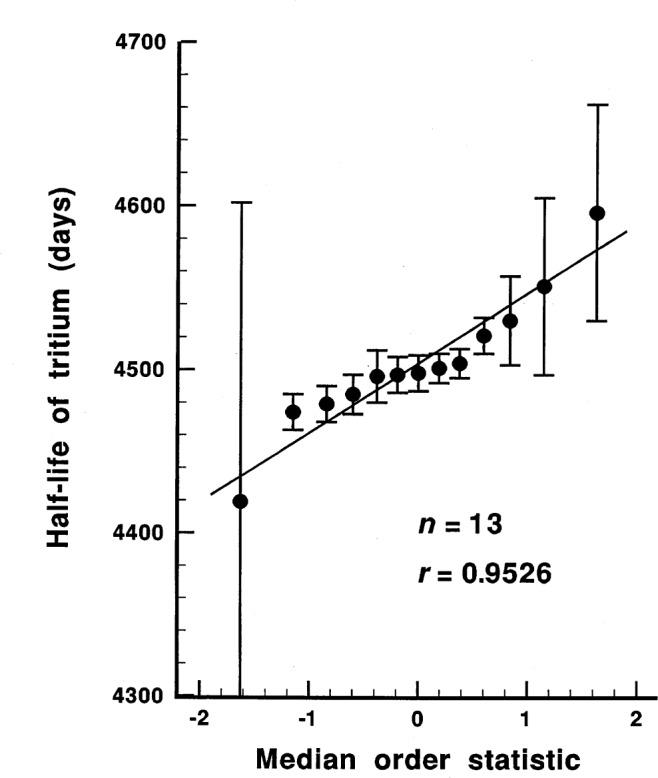
Normal probability plot for the *n* = 13 data set. The abscissa is the median order statistic from a normal *N*(0,1) distribution as given by Filliben [29]. The test statistic *r* is the normal probability plot correlation coefficient (i.e., the correlation coefficient for the linear regression line that is shown).

We tested the data, both *n* = 11 data points and *n* = 13 data points, for normality (strictly speaking, for not non-normality) using the probability plot correlation coefficient test for normality developed by Filliben [29]. The results are shown in Figs. 1 and 2. The test statistic, *r*, is the normal probability plot correlation coefficient. For *n* = 11, *r* = 0.961, and the probability that the data are normally distributed is approximately 0.3. Based upon this probability, the assumption that the data are normally distributed would usually be accepted. For *n* = 13, *r* = 0.952, and the probability that the data are normally distributed is approximately 0.15. The assumption that the data are normally distributed is now more marginal, although typically a probability of less than 0.10, or perhaps even less than 0.05, is required before rejecting the hypothesis of normality.

We have included all 13 data points in Table 2. Because of the marginally normal distribution of the data points for *n* = 13, the statistical calculations were carried out with *n* = 11 and with *n* = 13 to see if there was any significant difference in the results.

## 5. Data Evaluation Methods

The values shown in Table 2 were evaluated using three statistical methods, both without (*n* = 11) and with (*n* = 13) the first and last entries [8,14]. The results are shown in Table 3. The evaluation methods used were as follows (*u* denotes the estimated standard uncertainty):

- Determine the median and the estimated standard deviation of the median. This method is very robust with regard to outliers. We have used the method of Müller [30] to obtain the estimated standard deviation of the median. (The Müller paper appears in this issue of the *Journal* immediately following this paper.)
- Determine the weighted mean using equal weights of *w_i_* = (1/*u_i_*^2^)_avg_ and the estimated standard deviation of this mean. The equally-weighted mean (usually called the unweighted mean if using weights *w_i_* = 1) is unaffected by the individual stated uncertainties and does not reflect the fact that measurement capabilities have improved over time. The concern with this method is that the results may be influenced too much by the values with stated uncertainties higher than (*u_i_*)_avg_. The estimated mean is not affected by the actual values of the weights, as long as all of the weights are equal. The reason that we set the weights equal to the average value of 1/*u_i_*^2^ is so that we can calculate the estimated standard deviation of the mean in the same ways that we use with method C.
- Determine the weighted mean using weights *w_i_* = (1/*u_i_*^2^) and the estimated standard deviation of this mean. This method minimizes the estimated variance and emphasizes the stated uncertainties very strongly. The concern with this method is that the results may be influenced too much by the values with the smallest stated uncertainties, some of which may be underestimated.

## 6. Formulas Used

The estimated standard deviation of the median was computed using the method of Müller [30]:

$$S_{\text{median}}=\frac{1.858\;\mathrm{MAD}}{\sqrt{n-1}},$$ (1)

where

*MAD* is the mean absolute deviation from the median, and
*n* is the number of data points (11 or 13).

The estimated mean, denoted by *m*, was computed from

$$m=\frac{\Sigma w_{i}x_{i}}{\Sigma w_{i}},$$ (2)

where the *x_i_* are the experimentally-determined values of the half-life of tritium shown in Table 2 and the *w_i_* are the corresponding assigned weights.

The estimated variance of the mean, denoted by *s_m_*^2^, was computed as

$${s_{m}}^{2}=\frac{\Sigma w_{i}{\left(x_{i}-m\right)}^{2}}{v\Sigma w_{i}},$$ (3)

where *v* = *n* − 1 is the degrees of freedom. The estimated standard deviation of the mean, denoted by *s_m_*, is the square root of the estimated variance of the mean.

If the quantity

$$\frac{\Sigma w_{i}{\left(x_{i}-m\right)}^{2}}{v}=\frac{\chi^{2}}{v}=R^{2}$$ (4)

is equal to one, then Eq. (3) reduces to simply

$${S_{m}}^{2}=\frac{1}{\Sigma w_{i}}.$$ (5)

This will be the case if the weights used are equal to the inverse of the actual variances (i.e., if each *w_i_* = 1 / (*x_i_* − *m*)^2^).

We have never seen an experimental data set for which Eq. (4) was actually equal to one (certainly not any data set where the uncertainty of each data point was evaluated by a different experimenter). None-theless, Eq. (5) is often used, perhaps because of computational convenience. The reduced chi-squared, *χ*^2^ / *v*, and the Birge ratio, *R*, are measures of the degree to which the weights used are, in fact, equal to the inverse of the actual variances. If the reduced chi-squared and the Birge ratio are significantly larger than one, then the data are suspect and it is likely that at least some of the weights are overestimated (i.e., at least some of the variances are underestimated). Likewise, if the reduced chi-squared and the Birge ratio are significantly smaller than one, it is likely that at least some of the weights are underestimated.

For example, if we use the *n* = 11 data set with the author’s equivalent standard uncertainties, then we get *m* = 4496.3 d and

$$\frac{\Sigma w_{i}{\left(x_{i}-m\right)}^{2}}{v}=\frac{\chi^{2}}{v}=R^{2}=18.2.$$

Thus Eq. (5) underestimates the variance of the mean by a factor of 18.2 (underestimates the standard deviation of the mean by a factor of 4.3). This is the result of the very low uncertainties in Refs. [13] and [19].

If we use the reevaluated standard uncertainties shown in Table 2 for the *n* = 11 data set, then we get *m* = 4496.7 d and

$$\frac{\Sigma w_{i}{\left(x_{i}-m\right)}^{2}}{v}=\frac{\chi^{2}}{v}=R^{2}=1.61.$$

Eq. (5) now underestimates the variance of the mean by only a factor of 1.61 (underestimates the standard deviation of the mean by a factor of 1.3).

If we use the reevaluated standard uncertainties shown in Table 2 for the *n* = 13 data set, then we get *m* = 4497.0 d and

$$\frac{\Sigma w_{i}{\left(x_{i}-m\right)}^{2}}{v}=\frac{\chi^{2}}{v}=R^{2}=1.5.$$

It is our experience that most experimenters tend to underestimate their own uncertainties, so that Eq. (5) almost always gives a smaller value than Eq. (3). In Table 3 we present the estimated standard deviations of the mean calculated using Eqs. (3) and using Eq. (5). As expected, the values calculated using Eq. (5) are significantly smaller than the values calculated using Eq. (3).

## 6. Discussion of Results

### We can not emphasize strongly enough that estimated uncertainties have large uncertainties

We used the half-lives and the reevaluated standard uncertainties shown in Table 2 to calculate the values shown in Table 3. The estimated standard deviations of the mean vary by a factor of 2 or more (the estimated variances of the mean by a factor of 4 or more), depending upon the equation (and the inherent assumptions) used to calculate them. We think that it is important for experimenters, and reviewers as well, to explicitly state how each estimated uncertainty was obtained.

Each adopted value resulting from this evaluation is the grand average of the results obtained using methods A, B, and C with *n* = 11 and with *n* = 13 (see Table 3 and Sec. 5, Data Evaluation Methods). Whether based upon 11 data points or 13 data points, the average value obtained for the half-life of tritium is almost exactly the same (4499.3 d and 4499.6 d). The average standard uncertainty (estimated standard deviation of the mean) is slightly larger with *n* = 13 than with *n* = 11 (8.7 d vs 7.5 d).

## 7. Comparison with Other Evaluations

Others have also compiled and evaluated the half-life of tritium. The first compilation of nuclear data for radioactive isotopes was published by Fea in 1935 [31]. In 1940, Livingood and Seaborg [32] published the first in a series of compilations [32,33,34,35,36,38,44,48] that has become the Table of Isotopes, now in its eighth edition. The first compilation of adopted or recommended values was that of Goldstein and Reynolds [37] in 1966. Estimated uncertainties were given as ranges (<1 %, 1 % to 5 %, >5 %). Adopted values, although not called that, were also given in Refs. [38] and [44], but no uncertainty estimates were provided.

Table 4 is a summary of the evaluations that have been published since 1960. As more independent measurements of the half-life of tritium have been reported, the published adopted or recommended values have converged. There seems to be very good agreement among the four most recent evaluations with regard to the adopted half-life of tritium and, except for Ref. [48], with regard to the adopted standard uncertainty. The half-life and uncertainty given in Ref. [48] were taken from the Evaluated Nuclear Structure Data File (ENSDF) [46] and the uncertainty appears to be too high by about a factor of two. We are still trying to determine the origin of these values, which appear to have been in ENSDF since about 1987.

## 8. Final Recommended Value

Our final recommended value for the half-life of tritium is the average of the adopted values from the four most recent evaluations, (4500 ± 8) d, where 8 d corresponds to one standard uncertainty. See Table 4.

## Figures and Tables

**Table 1:** Experimentally-determined values of the half-life of tritium reported between 1936 and March 2000, arranged in chronological order

Ref.	Year	Author(s)	Measurement method	Half life (years)	Stated uncertainty (years)	Meaning of the stated uncertainty	Comments
[2]	1936	McMillan	Ionization current	>10	None	No uncertainty	Followed decay of radiation from irradiated beryllium for 4 months. Omitted: limit only.
[4]	1940	Alvarez and Cornog	Beta counting	0.41	0.11	Not given	One sample followed for 80 d. Chamber had diffusion losses. Omitted: updated in [6].
[6]	1940	Alvarez and Cornog	Beta counting	>10	None	No uncertainty	One sample followed for 5 months in new chamber. Omitted: limit only.
[7]	1940	O’Neal and Goldhaber	Beta counting	31	8	Not given	Counted tritium from irradiated lithium metal. Omitted: outlier.
[8]	1947	Novick	Helium-3 collection	12.1	0.5	Not given	Two samples; accumulation times of 51 d and 197 d.
[9]	1947	Goldblatt et al.	Ionization current	10.7	2.0	Not given	Hydrogen+tritium in ionization chamber over 18 d. Omitted: outlier.
[10]	1949	Jenks et al.	Helium-3 collection	12.46	0.20	Not given	Repeated measurements every two weeks until stable. Omitted: updated in [11].
[11]	1950	Jenks et al.	Helium-3 collection	12.46	0.10	Probable error^a^	Four measurements over 206 d.
[12]	1951	Jones	Beta counting	12.41	0.05	Probable error^a^	Measurement of specific activity of tritium gas.
[13]	1955	Jones	Helium-3 collection	12.262	0.004	Not given	Two samples; accumulation times of 578 d and 893 d.
[14]	1958	Popov et al.	Calorimetry	12.58	0.18	Not given	One sample; 21 measurements over 13 months.
[15]	1963	Eichelberger et al.	Calorimetry	12.355	0.010	Probable error^a^	Two samples measured over four years. Omitted: updated in [17]
[16]	1966	Merritt and Taylor	Beta counting	12.31	0.13	Not given	Five gas counting measurements over 13 years.
[17]	1967	Jordan et al.	Calorimetry	12.346	0.002	Probable error^a^	Five samples; 266 measurements over 6 years. Omitted: updated in [19].
[18]	1967	Jones	Helium-3 collection	12.25 12.31	0.08 0.42	99.7 % confidence limits	Two samples; accumulation times of 450 d to 800 d. Only the first value is usually quoted.
[19]	1977	Rudy and Jordan	Calorimetry	12.3232	0.0043	95 % confidence limits	Eight samples; 1353 measurements over 16 years.
[20]	1980	Unterweger et al.	Beta counting	12.43	0.05	1 standard uncertainty	Two sets of gas counting measurements 18 years apart. Omitted: updated in [26].
[21]	1987	Budick et al.	Bremsstrahlung counting	12.29	0.10	Not given	Two samples of tritium+xenon gas measured over 320 d. Omitted: updated in [25].
[22]	1987	Oliver et al.	Helium-3 collection	12.38	0.03	1 standard uncertainty	Fifteen samples, each with accumulation times of 1 year to 2 years.
[23]	1987	Simpson	Beta counting	12.32	0.03	1 standard uncertainty	Tritium implanted in Si(Li) detector measured over 5.5 years.
[24]	1988	Akulov et al.	Helium-3 collection	12.279	0.033	1 standard uncertainty	Five series of measurements over 846 d.
[25]	1991	Budick et al.	Bremsstrahlung counting	12.31	0.03	1 standard uncertainty	Two samples of tritium+xenon gas measured over 5.5 years.
[26]	2000	Unterweger and Lucas	Beta counting	12.33	0.03	1 standard uncertainty	Three sets of gas counting measurements over 38 years.

a The probable error, *PE*, is the deviation from the population mean, *μ*, such that 50 % of the observations may be expected to lie between *μ* − *PE* and *μ* + *PE*. For a normal distribution, the probable error can be converted to the standard deviation by multiplying by 1.4826.

**Table 2:** Experimentally-determined values of the half-life of tritium reported between 1947 and March 2000, arranged in order of increasing half-life. The values from references [2,4,6,7,9,10,15,17,20,21] have been omitted. We have also reevaluated the uncertainties

Ref.	Year	Author(s)	Measurement method	Half life (days)	Standard uncertainty, *u* (days)	Comments
[8]	1947	Novick	Helium-3 collection	4419	183	Author’s stated uncertainty.^a^
[18]	1967	Jones	Helium-3 collection	4474	11	Author’s equivalent standard uncertainty.
[13]	1955	Jones	Helium-3 collection	4479	11	Our estimate of the standard uncertainty. (The author’s stated uncertainty gives *u* = 1.5 d.)^a^
[24]	1988	Akulov et al.	Helium-3 collection	4485	12	Author’s stated standard uncertainty.
[16]	1966	Merritt and Taylor	Beta counting	4496	16	Our estimate of the standard uncertainty. (The authors’ stated uncertainty gives *u* = 47 d.)^a^
[25]	1991	Budick et al.	Bremsstrahlung counting	4497	11	Authors’ stated standard uncertainty.
[23]	1987	Simpson	Beta counting	4498	11	Author’s stated standard uncertainty.
[19]	1977	Rudy and Jordan	Calorimetry	4501	9	Our estimate of the standard uncertainty. (The authors’ stated uncertainty gives *u* = 0.79 d.)
[26]	2000	Unterweger and Lucas	Beta counting	4504	9	Authors’ stated standard uncertainty.
[22]	1987	Oliver et al.	Helium-3 collection	4521	11	Authors’ stated standard uncertainty.
[12]	1951	Jones	Beta counting	4530	27	Author’s equivalent standard uncertainty.
[11]	1950	Jenks et al.	Helium-3 collection	4551	54	Authors’ equivalent standard uncertainty.
[14]	1958	Popov	Calorimetry	4596	66	Authors’ stated uncertainty.^a^

a The meaning of the author’s stated uncertainty was not given. The value shown assumes that the stated uncertainty is one standard uncertainty.

**Table 3:** The half-life of tritium and the estimated standard deviation of the mean calculated using three statistical evaluation methods with *n* = 11 and with *n* = 13. *u* denotes the estimated standard uncertainty. The half-lives and uncertainties used are shown in Table 2. See Table 4 for our final recommended values.

Method of statistical evaluation	Half-life (days)		Standard deviation (days)	
	*n* = 11	*n* = 13	*n* = 11	*n* = 13
A. Median	4498.0	4498.0	11.2	10.2
B. Weighted mean using Eq. (2)				
with *w_i_* = (1/*u* ^2^*i*)_avg_	4503.3	4503.9		
Standard deviation of the mean				
using Eq. (3)			6.9	11.6
using Eq. (5)			(3.6)^a^	(3.6)^a^
C. Weighted mean using Eq. (2)				
with *w_i_* = (1/*u* ^2^*i*)	4496.7	4997.0		
Standard deviation of the mean				
using Eq. (3)			4.5	4.4
using Eq. (5)			(3.6)^a^	(3.6)^a^
Average of A, B, and C	4499.3	4499.6	7.5	8.7
Adopted value resulting from this evaluation			8	
(See Table 4 for our final recommended values)	4499			

a Values in parentheses are not included in the average.

**Table 4:** Values of the half-life of tritium and the standard uncertainty that have been adopted or recommended in evaluations published since 1960.

Ref.	Year	Author(s)	Adopted Half life (days)	Standard uncertainty (days)	Comments
[37]	1966	Goldstein and Reynolds	4492	<45	Origin of this value not stated.
[38]	1967	Lederer et al.	4492	Uncertainty not given	Unspecified combination of [11,13,14].
[39]	1970	Martin and Blichert-Toft	4511	4	Unspecified combination of [8,11,12,13,14,17,18].
[40]	1970	Sher	4493	15	Weighted mean of [11,12,13,14,16,17,18].
[41]	1972	Keeton	4506	1.5	Private communication from Jordan [17,19] with increased uncertainty.
[42]	1973	Piel	4483	17	Weighted mean of [13,15].
[43]	1978	Raman et al.	4503	5	Weighted mean of [8,11,12,13,14,15,17,18].
[44]	1978	Lederer and Shirley	4503	Uncertainty not given	Weighted mean of [11,13,14,17].
[45]	1981	Kocher	4485	11	Apparently from ENSDF [46], as of October 1977.
[47]	1990	Holden	4499	8	Average of weighted means for each method.
[48]	1996	Firestone and Shirley	4503	22	Taken from ENSDF [46]. The origin of these values has not been determined.
[49]	1999	Bé et al.	4500	7	See Ref. [50] for details of the tritium half-life evaluation.
This work	2000	Lucas and Unterweger	4499	8	Adopted value from Table 3.
Our final recommended value			4500	8	Average of adopted values from Refs. [47,48,49] and Table 3.^a^

a Our final recommended value for the standard uncertainty is the average of three of the four most recent adopted uncertainties. The uncertainty given in Ref. [48] was omitted because it appears to be too high by about a factor of 2.
